# No one’s discussing the elephant in the room: contemplating questions of research impact and benefit in Aboriginal and Torres Strait Islander Australian health research

**DOI:** 10.1186/s12889-015-2052-3

**Published:** 2015-07-23

**Authors:** Roxanne Bainbridge, Komla Tsey, Janya McCalman, Irina Kinchin, Vicki Saunders, Felecia Watkin Lui, Yvonne Cadet-James, Adrian Miller, Kenny Lawson

**Affiliations:** The Cairns Institute, James Cook University, PO Box 6811, Cairns, Queensland 4870 Australia; Midwifery and Nutrition, School of Nursing, James Cook University, 1 James Cook Drive, Townsville City, Queensland 4811 Australia; Indigenous Centre, James Cook University, PO Box 6811, Cairns, Queensland 4870 Australia; Indigenous Centre, James Cook University, James Cook Drive, Townsville City, Queensland 4811 Australia; Indigenous Research Unit, Griffith University Nathan Campus, 170 Kessels Road, Nathan, Queensland 4111 Australia; Centre for Chronic Disease Prevention, James Cook University, PO Box 6811, Cairns, Queensland 4870 Australia

**Keywords:** Aboriginal and Torres Strait Islander, Indigenous, Health, Research benefit, Research impact, Research translation

## Abstract

**Background:**

There remains a concern that Indigenous Australians have been over-researched without corresponding improvements in their health; this trend is applicable to most Indigenous populations globally. This debate article has a dual purpose: 1) to open a frank conversation about the value of research to Indigenous Australian populations; and 2) to stimulate ways of thinking about potential resolutions to the lack of progress made in the Indigenous research benefit debate.

**Discussion:**

Capturing the meaning of research benefit takes the form of ethical value-oriented methodological considerations in the decision-making processes of Indigenous research endeavours. Because research practices come from Western knowledge bases, attaining such positions in research means reconciling both Indigenous and Western knowledge systems to produce new methodologies that guide planning, evaluating and monitoring of research practices as necessary. Increasingly, more sophisticated performance measures have been implemented to ensure academic impact and benefits are captured. Assessing societal and other non-academic impacts and benefits however, has not been accorded corresponding attention. Research reform has only focussed on research translation in more recent years. The research impact debate must take account of the various standards of accountability (to whom), impact priorities (for whom), positive and negative impacts, and biases that operate in describing impact and measuring benefit.

**Summary:**

A perennial question in Indigenous research discourse is whether the abundance of research conducted; purportedly to improve health, is justified and benefits Indigenous people in ways that are meaningful and valued by them. Different research stakeholders have different conceptions of the value and nature of research, its conduct, what it should achieve and the kinds of benefits expected. We need to work collaboratively and listen more closely to the voice of Indigenous Australians to better understand, demonstrate and measure health research benefits. The authors conclude that as an imperative, a systematic benefit assessment strategy that includes identification of research priorities and planning, monitoring and evaluation components needs to be developed and implemented across research projects. In Indigenous health research, this will often mean adopting a benefit-led approach by changing the way research is done and preferencing alternative research methodologies. As a point of departure to improving impact and reaching mutually beneficial outcomes for researchers and partners in Indigenous health research, we need to routinise the assessment of benefit from outset of research as one of the standards toward which we work.

## Background

In Aboriginal and Torres Strait Islander health research, research benefit is broadly defined as any elements of research that are advantageous or good; such as strengthening capacities, opening opportunities or improving health outcomes that progress the interests that are valued by Aboriginal and Torres Strait Islander (respectfully hereafter Indigenous) people [1]. Historically however, health research has not served Indigenous Australians well [2]; this has generally been the case for Indigenous people in colonised nations globally [3]. Despite relatively small demonstrable achievements more recently, there still remains concerns that Indigenous Australians have been over-researched without corresponding improvements in health outcomes: “despite all the research and medical interventions spaning decades, improvements – where they occur – are incremental and trend up at a slower rate than for non-Indigenous Australians” [4].

It is not surprising then that Indigenous people continue to question the value of research, particularly in terms of accomplishing benefits and social change that accrue as a result of research. Key reasons for the poor translation of research findings into indicators of social change or benefits has been because in large measure, research has been controlled by non-Indigenous people, and conducted ‘on’ Indigenous people; and been strongly biased toward the incentives of the colonising society [2]. Despite some reform aligned with Indigenous political agitation and environments over time, an underlying mistrust of researchers and associated research activities still persist in Indigenous populations [2]. Anecdotally, poorly undertaken government projects and consultations about public policy implementation has also led Indigenous Australian communities to categorise these activities as research; often adding to a perception that research has not benefited communities.

For decades, critiques of research practices have been submitted by Indigenous people and failure on the part of researchers to address these criticisms in any substantial way have been directly attributed to continuing poor research outcomes from Indigenous health research. Key criticisms include: 1) a focus on descriptive research that has done little more than document the extent of Indigenous Australian disadvantage and which has made minimal impact in terms of improving health conditions for Indigenous people [5]; 2) research has been considered “seriously damaging and harmful” and “insensitive, intrusive and exploitative” [6–9]; 3) research practices perpetuate the exploitative history of Australian colonialism and objectivise Indigenous people [10–12]; 4) a large proportion of research into Indigenous health has been designed to serve the academic, political or professional needs of researchers [9]; and 5) despite identifying some clear research priorities for improving health outcomes, such as the significance of the social determinants, these have not been translated into action.

Such poor research outcomes, perceptions of no benefit and an ongoing underlying mistrust of research on the part of Indigenous Australians leave little doubt that contemporary agendas for conducting Indigenous research require new regimes to realise change and have relevance to the lives of the people with whom we work [13, 14]. New research regimes unquestionably lie at the confluence of the ethics of practice, the practice of ethics and Indigenous and Western knowledges and value systems. Priority setting for benefit is an imperative that must underpin change agendas across all research endeavours; and particularly those conducted with goals of increasing the value of research to Indigenous Australian health. Such benefits include improvements in health, participation and empowerment; strengthened capacity; and academic outputs (papers, grants, research students). With the concept of research benefit proposed as a method that can potentially effect a significant shift in the current way of doing or thinking about research as a point of reference, in this debate article, we pose an ethical question similarly asked by Australia’s National Health and Medical Research Council (NHMRC) [1] some 15 years ago: how can the interests of research and researchers be integrated with the values, expectations and cultures of Aboriginal and Torres Strait Islander communities for increased benefit in changing climates of social, economic and political accountability? Without a game-changer interjected into this space, we will again be confronted with the same research question in another 15 years’ time. This article was designed for two purposes: 1) to open a frank conversation on the value of research to Indigenous Australian populations; and 2) to stimulate ways of thinking about potential resolutions to the lack of progress in the Indigenous research benefit debate by using the conclusions to inform the development of a preliminary framework for thinking about research benefit. It was guided by the following research questions:

- What is research and what is its value?
- What is research benefit and how it is demonstrated?
- Whose perspective is represented?
- What are some of the opportunities and challenges in working to maximise research benefit including the approaches researchers have taken to increase research benefit in Indigenous health research? and
- How can research benefit be measured?

## Discussion

### What is research and what is its value?

There are variances in the nature of research and thus its meaning, purpose and value. There is agreement nevertheless, that the primary intent of research is to systematically generate new knowledge. To unpack its meaning, we looked at a definition of research to which Australia’s NHMRC submits and that originated from the United Kingdom Research Assessment Exercise. It proposes that research “… includes work of direct relevance to the needs of commerce, industry, and to the public sectors; scholarship; the invention and generation of ideas; images, performances, artefacts or design where these lead to new or substantially improved insights; and the use of existing knowledge in experimental development to produce new or substantially improved materials, devices, products, and processes including design and construction” [15]. Inferences drawn from this definition indicate that whatever form research takes, it must be useful and directly relevant to the advancement of knowledge for societal progress and human health and flourishing; and designed to produce innovations that have impact in such contexts. For these reasons, research is necessarily intertwined with: 1) societal ethics, whereby research benefits must outweigh any harm; and 2) values that are temporally and contextually open to interpretation and change.

Despite variance in perceptions of research, the value of health research to individuals and society is indisputable [2]. But it should be noted that research is a means to an end, rather than an end itself; and that alone it will not necessarily derive benefits or be of value. But the generation of new knowledge contributes to improvements in health care and public health by providing information about disease trends, risk and protective factors, patterns of care and health care costs, and developing new therapies and treatments, and assessing the effectiveness of health interventions. Research alone does not generate benefits. The degree to which health research is valuable and makes substantive contributions to society however, depends on its nature, quantity and quality [16] and whether action processes or translational work occurs across the life, or at the back-end of research [17]. In terms of the latter, it is clear that “society can reap the benefits of successful research studies only if the results are converted into marketable and consumable products (e.g., medicaments, diagnostic tools, machines, and devices) or services” [18]. But achieving research benefit involves time-oriented processes; for instance, there is reportedly “an average time-lag between research funding and impacts on health provision of around 17 years” [19]; for example, the translation of a research discovery in a biomedical lab to day-to-day clinical practice [20].

### What is research benefit and how is it demonstrated?

In a policy context, Indigenous Australian people define research benefit as “the establishment or enhancement of capacities, opportunities or outcomes that advance the interests of Aboriginal and Torres Strait Islander peoples and that are valued by them” [1]. Here, benefit is demonstrated by reciprocity [1]; where reciprocity refers to the Indigenous value of mutual obligation and which aims “to achieve an equitable distribution of resources, responsibility and capacity and to achieve cohesion and survival of the social order” [1]. Translation of reciprocity into a research context infers “inclusion, recognition of partners’ contributions, and ensuring that research outcomes include equitable benefits … for communities or individuals” [1]. For researchers, reciprocity requires honouring research partnerships by demonstrating benefit; as defined and valued by the community according to their priorities and which contributes to their cohesion and survival [1].

While capturing the meaning of research benefit necessarily takes the form of ethical value-oriented methodological considerations in the decision-making processes of research endeavours, there are also conceptual issues to be resolved in distinguishing the difference between the terms that speak to benefit. For example, in using the terms ‘research impact’ and ‘research benefit’, some people use benefit and impact as discrete terms; some use the two terms interchangeably; and different disciplinary perspectives direct various usage. In this article, the authors worked from the assumption that the two terms are distinct from each other, while at times interrelated and interdependent. ‘Research impact’ is considered to be any area of influence flowing from the research endeavour including those that flow from research processes; these can be both positive and negative. ‘Research benefit’ on the other hand, flows from areas of impact. This benefit can be intended or unintended, inside or outside the immediate research environment, direct or indirect, tangible or intangible, immediate, short-term or longer-term; but benefit must be positively oriented and represented as elements of value derived from research.

There are apparent inconsistencies regarding the meaning of research impact and benefit, and therefore also disagreement about how it can be measured. The Australian Research Council (ARC) [21] defines research impact as “the demonstrable contribution that research makes to the economy, society, culture, national security, public policy or services, health, the environment, or quality of life, beyond contributions to academia”. It includes, but is not limited to, an effect on, change or benefit to: “the activity, attitude, awareness, behaviour, capacity, opportunity, performance, policy, practice, process or understanding of an audience, beneficiary, community, constituency, organisation or individuals in any geographic location whether locally, regionally, nationally or internationally”. A group from the United Kingdom who have been particularly active in the field of research impact, the London School of Economics (LSE) Public Policy Group [22] assert that it is “an occasion of influence and hence it is not the same thing as a change in outputs or activities as a result of that influence, still less a change in social outcomes”. They suggest two broad areas of impact from which benefits are derived: 1) Academic impacts - influences upon actors in academia or universities e.g., as measured by citations in other academic works; and 2) External impacts - influences on actors outside higher education, in business, government or civil society e.g., as measured by references in government documents. Other UK researchers have expanded on those areas of impact. For instance, Kuruvilla, Mays, Pleasant & Walt [23] present an evidence-based assessment of the nature of impact and its associated benefits. In developing their health research impact framework, based on a literature review and empirical analysis of selected London School of Hygiene and Tropical Medicine research projects, they identified four key areas of research impact: 1) research-related, 2) policy, 3) services: health and intersectoral, and 4) societal impacts. They claim that the four categories are not exhaustive or mutually-exclusive, and are at times interdependent and overlapping. Yet others have suggested that these elements too often operate in silos [24] or have a stronger focus on research-related impact and benefits which largely influence actors in academia or universities and thus often represent the key benefits measured [23]. For example, Excellence in Research for Australia has been introduced by the Commonwealth Government to provide more rigorous measures of research quality across the higher education system [21]. It aims to assess research quality using a combination of metrics focused on researchers, research outputs, research income, esteem and applied measures. These indicators of productivity are quantifiable, but generally do not translate into robust measures as valued by end-users of research. We take the view that academic benefits and other contributions need not be mutually exclusive and that the various elements should concurrently interact.

The debate around research impact and benefit nevertheless, is ever-progressing and reflected in recent planning by the Commonwealth Government of Australia [25]. The government aim is to develop a mechanism by which the broader economic, social and environmental benefits resulting from government research investment, including the benefits arising from university-based research can be assessed [25]. Driving this change is an attempt to broaden the scope of research impact and benefit because although the benefits from research have been under scrutiny since the 1990s [26], the primary focus was on benefits flowing from the impacts of research on academia and scientific knowledge [17].

### Reform in indigenous health research

Despite major developments driving changes in Indigenous health research methodologies and practices in recent decades – like the establishment of the Lowitja Institute, Australia’s only Indigenous-controlled health research organisation [4] and the Aboriginal Health and Medical Research Council of New South Wales - Indigenous people are still concerned about the ethics of research, including how research is developed, done and whether it has tangible benefits as determined them [27]. In leading reform, such institutions have applied a decolonising lens to think about, and implement broader Indigenous research strategies that foreground Indigenous knowledge, values and practices [28]. A decolonising approach requires a power shift in research relations and is embedded in a social justice perspective. It entails for instance, the privileging of Indigenous voices and epistemologies in collaborative research endeavours. For example, Lowitja Institute has embraced collaborative research approaches and lead the way in funding knowledge exchange and translation that ensures research is priority-driven and that evidence-based research findings are used to improve Indigenous health practice and well-being in sustainable ways [29]. The Institute has also actively pursued identifying the future demands for Indigenous health research to ensure the relevance of research into the future and thus maximise potential benefits.

Indigenous research reform has however, focussed predominantly on process issues; and only in more recent years has research translation to maximise benefits become a priority. As an example, the reporting of research benefit and research translation in Indigenous health is miniscule in Australia. A systematic search of four key databases (Medline, Scopus, Informit and Cinahl) using the terms ‘health’, ‘research benefit’, ‘research impact’, ‘Aborigin*’, ‘Indigenous’, ‘research translation’ and ‘knowledge translation’ and with the limits of Australia and 1995 – 2014 returned a total of 59 hits. Of these, only 2 publications considered, but did not focus on, research benefits and translation.

Indigenous researchers and scholars however, have long sought to reform mainstream Indigenous health research methodologies and practices [28, 30–32]. With the goal of achieving measurable benefits as defined by Indigenous people, they have led these reform agendas and created Indigenous methodologies that have as one of their key defining features sustainable benefit. These reform agendas align with Indigenous aspirations of empowerment and self-determination. As such they have an emancipatory imperative, political integrity and privilege Indigenous voices [30] by recognising Indigenous ways of knowing, ways of being and ways of doing [31, 32]. Simultaneously, funding bodies such as the NHMRC [1] have produced ethical guidelines for Indigenous research and prioritised research funding based on ethical community engagement and research relevance, that is identified and driven by Indigenous communities. In response, many non-Indigenous researchers have amended their approaches to research by nurturing closer partnerships with Indigenous research stakeholders and reoriented to participatory action research methodologies to increase the appropriate application of research evidence in policy and practice [11, 33, 34]. Funding bodies have also revised their practice; for instance, the CEO of Australia’s NHMRC advises that the assessment and ranking of funding applications are also determined based on the importance of an idea as judged according to its ability for impact on knowledge development and beyond [35].

Apart from the call from Indigenous people to increase research benefits, in the broader policy context of research there has been a demand for more accountability in public spending across all sectors. In times of tight fiscal government environments, governments and others are increasingly shifting toward research-based evidence as a way of maximising efficiency and efficacy in policy and public service delivery. Nevertheless, there has been increased health research expenditure. In Australia and most OECD countries, expenditure on health care, both public and private, has been rising faster than Gross Domestic Product (GDP) over the past decade [36]. In Australia, research and development expenditure in health as a proportion of total spending on other health care increased from 11.0 % in 2001–2002 to 17.5 % in 2011–2012 [36]. For example, the Australian Government invested in health research with expenditure increasing from $783 million in 2009–10 to $798 million in 2011–12 [36]. Increased expenditure means a justifiable expectation of increased returns on investment. The role of publicly-funded research organisations, as well as public funding arrangements for research has been redeveloped to reflect this position [21]. That is, research must be now focused to “maximise resource effectiveness, quality and impact and its ability to meet Australia’s strategic needs” [21].

Additional to these reforms, Thomas, Bainbridge & Tsey [2] more recently suggested that health equity for Indigenous Australians is also more likely to be realised through “improved research processes and ethical frameworks to guide and keep accountable researchers and research organisations, alongside the development of methods to assess research impact and benefit for Indigenous people”. Others have also explored these issues. For instance, research implementation fidelity has been raised as an concern [37, 38] and Humphrey [11] has raised questions about the extent of Indigenous research transformation and its potential to bring “about fundamental change in research practice” vis-à-vis “being sidelined into too great a reliance on written guidelines and positive rhetoric”.

However, and despite evidence of changing trends in Indigenous health research, there has been little focus on ways of systematically determining whether a particular piece of research is of benefit to Indigenous people. Indeed, there “is no systematic process of measuring the broader economic, social and environmental benefits of [any] publicly funded research” at all [25]. The Australian Government however, is focussed on improving accountability and the benefits arising from university-based research and is working toward building a framework to measure research benefit in a systematic way [25]; this is not surprising given that only one-tenth of research funding leads to measurable impact and benefits [17]. For example, a survey of 76 research and development managers in United States firms were asked to make estimations of the proportion of products or processes that could not have been developed in the last 10 years without academic research. Calculations were made at only 10 % for new products and 11 % for new processes [17]. Herein, lays further evidence to compel researchers toward working to achieve a more coordinated, coherent approach that maximises benefits from funding investment in research.

### Whose perspective?

The preceding definitions demonstrated the variable contexts in which people distil the meaning of research benefit and the hefty weighting that academic impact and related benefits hold. The delineation of benefit and its explicit priority setting is an important first step in its demonstration, achievement and measurement; and inevitably leads to questions about benefit from whose perspective. In contemplating questions of research benefit for Indigenous Australians, a number of circumstances come to mind that need to be well thought through. One outstanding issue to be considered when articulating an impact-benefit narrative is that although there are overlapping aspects, the concept of research benefit does not translate equally across Indigenous and non-Indigenous contexts or the different levels at which impact is manifest. Traditionally, impact and therefore benefits has been measured by peer accountability in academia, as opposed to social accountability, with academic productivity measures such as research income, bibliometrics and citations taking precedence.

As researchers, we are very familiar with grant proposals where benefits are expressed in terms of research users, beneficiaries, communication and expected impact; research project evaluations and reports that fundamentally provide accountability for funding investment; media information, research dissemination; estimations of research impact on policy and practice; and individual or institutional research impact submissions [23]. However, the research impact debate must take account, for instance, of the various standards of accountability (to whom), impact priorities (for whom), positive and negative impacts, and biases that operate in describing impact and measuring benefit [23].

Understanding how people value and interpret the benefits of research is critical. While academics talk about research impact, Indigenous people talk about ethical positions and ‘tangible’ benefits such as responses and solutions to issues studied in research projects that are meaningful to their lives. These benefits might include the development of historical artefacts and languages that preserve culture and benefit future generations and direct benefits such as the translation of any research knowledge into more immediate relevant and consumable products. However, tangible for Indigenous people can mean both tangible and intangible benefits when perceived from Western standpoints; intangibles might include cultural determinants and concepts of wellbeing such as identity, empowerment and control. The question posed is therefore: to what extent do current measures fulfil the articulation of benefit from the perspective of Indigenous people and organisations with whom researchers work; and how do we work toward mutual research outcomes? – as the NHMRC Chief Executive Officer states, benefit demonstrated in the academic context, while valuable, should be seen as “just a milestone on the road to real impact in improved health” [35].

### What approaches have researchers taken to achieve impact and increase benefit?

Research ethics guidelines such as those expounded by the NHMRC and other international ethics review boards emphasise community-driven and controlled participatory approaches to conducting research with Indigenous partners. However, without authentic uptake and implementation of these, and other collaborative research processes; along with contemplated action at the back-end of research (research translation), research effects are minimised. Silburn et al. [39] note the development of the Cooperative Research Centre for Aboriginal Health’s facilitated research development process as a mediating approach for improving ethical implementation of research processes and thus increase benefit. They advocate that changing the processes of research accordingly – from priority setting to research transfer is a necessary part of maximising research benefit for Indigenous people. In particular, they suggest completely re-modelling research processes to improve the likelihood of research use. These included: 1) Indigenous people becoming direct and active participants; 2) identifying and prioritising issues of relevance to Indigenous people; 3) incorporating Indigenous knowledges and perspectives in processes and findings; 4) reporting findings were meaningful; and 5) engaging all potential end-users from the outset.

Partnership approaches are amenable to achieving impact and benefits in Indigenous research contexts. This is because partnerships strengthen the relationship between researchers and research users and bring together different knowledges and expertise to accomplish more than what could be achieved alone; thus improving the prospect of benefit [34]. Importantly, adding reflective processes that encourage the development of shared understandings of the situation under study also invite opportunities to examine expectations and interpretations of benefit. Action research is favoured for its usefulness in implementing action and change throughout the research process. Action grounded in localised knowledge and implemented throughout the research process to maximise impact and refine planning for instance, was a priority for Yarrabah stakeholders in developing a community social and emotional wellbeing action plan [40]. Building Indigenous research capacity, another strategy implemented by the NHMRC, has been successful in increasing the numbers of Indigenous researchers through its population health capacity building grants [41]. These grants were introduced in response to a review of Australian public health research and designed to strengthen research workforce capacity that has demonstrated a potential for the delivery of best practices; in turn potentially contributing to better engagement and research outcomes [2]. However in contemplating capacity development efforts, again, the foci often reside in the academic arena while Indigenous lay knowledge and the ways in which it can assign meaning and inform action to reduce health inequities is often overlooked. Working at the interface of scientific and cultural knowledge is an important undertaking in striving for change and maximising benefit [42].

These developments speak to the implementation of new research. Increasingly, alternative approaches such as systematic reviews are being conducted to ascertain the effectiveness of Indigenous health programs without burdening Indigenous populations with more research. Through the conduct of reviews, concerns have been expressed about the over-emphasis on descriptive research rather than research evaluating interventions in Indigenous health [16, 37, 43–48]. These reviews have additionally identified what Paul et al. [43] describe as “the sorry state of the evidence base for improving the health of Indigenous populations”. Further to identifying the dominance of descriptive research, many reviews note the overwhelmingly paltry numbers and poor methodological quality of studies. Given that the degree to which health research is valuable depends on the nature, quantity and quality and fitness to purpose of available evidence [16]; more robust research designs are needed to improve outcomes. Journal editors too have noted this gap; for instance, the *MJA*’s editors flagged that Indigenous health research has been too “observational and deficit-focused, with a dearth of interventional studies” and have “even considered putting a moratorium on publishing the many observational studies” being submitted [49].

### How can research benefit be measured?

Defining research impact and benefits is difficult, but drawing a linear link between a particular research study and subsequent improvement in health, and measuring that impact, is an even more intrinsically difficult task [35, 50]. This situation is hampered at times by political processes that can occur between research conduct and the realisation of outcomes, and over which researchers might or might not have control. Assessing the benefits of research is a necessary activity and can be used for a number of different purposes; thus approaches to interpreting benefit can be made by measuring it both quantitatively and qualitatively, including economic evaluations. However, narrative (qualitative) approaches have been critiqued because they are subjective, expensive to implement and hard to compare across contexts. Nevertheless, what we measure is based on what we value most; and perhaps why the motivations of the dominant society toward academic measures have been prioritised. Research findings and thus benefits are filtered through people’s interests and beliefs [51]; these need to be negotiated and clearly defined for research stakeholders, accounted for from the outset of the research and aligned with the objectives and intended outcomes. Benefits also need to be reviewed progressively. The question thus becomes what kinds of benefits are expected, rather than whether researchers can create impact through a plausible narrative. In doing that, whose voices are heard in contemplating impact needs to be considered, as is the need to challenge the assumptions of researchers and others.

The current state of Indigenous research assessment embodies a Western cultural framework based on measurable units and quantifiable methods and indicators. As a result, biomedical and economic indicators of research assessment do not account for measurable aspects of community living and wellbeing that are not easily measured. These less easily measured aspects include intangible cultural heritage and wellbeing, Indigenous people’s worldviews, associations and relationships [52, 53]. An easily accessible example is the effects of research on the health and wellbeing of Indigenous people. But the concept of health and wellbeing for Indigenous people expands on the Western concept of health to also include socially and culturally-based understandings of life. According to the National Aboriginal Health Strategy [9], Indigenous health is not just the physical wellbeing of an individual, but the social, emotional, and cultural wellbeing of the whole community in which each individual is able to achieve their full potential [53, 54]. Thus health for Indigenous people is a multi-dimensional concept. It embraces all aspects of living, including those valued by Western societies such as the social determinants of health, while also stressing the importance of survival and flourishing in harmony with the environment.

While the reference to Indigenous wellbeing indicators in policy is made, Indigenous cultural heritage as a factor in the wellbeing of Indigenous people is not simultaneously recognised [53]. Instead, the foci is on: life expectancy as a measure of health and wellbeing; employment and income, opportunities for self-development, living standards and self-esteem as important for overall wellbeing; household and individual income as a determinant of economic and overall wellbeing; home ownership as an important element in improving Indigenous wellbeing; infant mortality as an indicator of the general health and wellbeing [53]. These indicators of health and economic standard of living for Indigenous people have no reference to Indigenous wellbeing as it is understood in international and local Indigenous contexts [54]. The exclusion of intangibles such as wellbeing occurs primarily because the concept is not valued by Western systems in the same way and is difficult to measure using standard scientific approaches. However further to that, it should be noted that such societal impact approaches are evolving and more research is required to develop the appropriate methods. Also required is political will on the part of policy makers to invest in developing the methods to quantify what is important rather than what is easy to quantify. The current case study approaches are presently just too expensive and subjective to contemplate system-wide. Nevertheless, an obvious gap exists in current indicator-based approaches to capture and measure impacts of research from Indigenous perspective. There is an urgent need for working with (rather than on) Indigenous people in order to “overcome the continuing embeddedness of colonising practices in reductionist indicator-based approaches” [55] and develop effective local Indigenous scale indicators to capture and measure intangible impacts and benefits of Indigenous research.

Gaining consensus about the quality and nature of evidence can be divisive [24]. Methodologically, less value is placed on Indigenous and local knowledge and narratives of change. However, inclusions of these are imperative in establishing meaningful and relevant indicators to measure impact; they are particularly important in assigning meaning to experiences of inequality, implementation and change processes and understanding the context in which such indicators should be developed. The importance of context is exemplified in the research impact model developed by Kuruvilla et al. [23] which supports a multi-level approach to assessing impact including research-related, policy, services: health and intersectoral and societal impacts. In each of these areas, descriptive elements are identified for modification or tailored to the context in which the measurement of impact will occur. While researchers are preparing to push the boundaries of research to contemplate a more rounded approach to assessing benefit, internationally, countries such as the UK are looking to economic evaluations. However, Australia is lagging behind in this regard; for example, the NHMRC does not request that people look at return for investment or value for money in their applications for funding. It is however evident in Cooperative Research Centre schemes where applications explicitly call for grant applicants to model and monitor the longer-term economic benefits of their research. This is another important area to be explored in planning the assessment of impact and benefits.

Silburn et al. [39] cite some of the many challenges faced in measuring the impacts of Indigenous health research. These are precisely the barriers to doing policy relevant impact/benefit measures globally. Challenges include: 1) attribution, such as the multitude of factors influencing health and health outcomes and the difficulties in attributing changes in outcomes to changes from factors; 2) identifying the variety of ways health research can influence outcomes; 3) the time lag between the outputs and potential outcomes; 4) the meaning ascribed to change may not be obvious (for example, more investment in a particular condition might mean that it has become more prevalent or more severe, or that there are more available diagnostic tests; 5) that links from research to impact are generally not linear; 6) difficulties in attributing economic value to aspects of health; 7) lack of records documenting how research is taken up by end-users; 8) some measures of change such as knowledge, develop incrementally and often result from multiple research projects across time; and 9) developing frameworks that account for the priorities of various different stakeholders (for instance, while measuring economic impact might be important for government; the same premium on one indicator is not likely to be valued by community members.

## Summary

Solid evidence underscores the ongoing advocacy of Indigenous people to engage research that can demonstrate tangible health benefits; much of it “reiterates continuing concerns from Aboriginal and Torres Strait Islander Peoples about poor consultation, lack of communication and infringement of deeply held values arising from cross-cultural insensitivity — despite researchers’ compliance with the legal requirements of ethical guidelines” [1]. Yet there is still little research examining what constitutes benefit from Indigenous Australian points of view. In times of change and challenge, when the value of research is questioned and funding structures are being revised, it is critically important to reflect on the ways in which value is attributed to research benefits from diverse perspectives and the processes in which we engage to achieve this. Better understanding this aspect of research is needed so that researchers and Indigenous communities can monitor and evaluate the benefits of research. Finding optimal ways of pushing the boundaries of research processes in which we invest our energies can resolve some of the challenges posed in this article.

A perennial question in Indigenous research discourse is whether the abundance of research conducted; purportedly to improve health, is justified and benefits Indigenous people in ways that are meaningful and valued by them. In other words, have there been meaningful and sustainable research improvements in the indicators of health and wellbeing as defined by Indigenous Australians? And, are the improvements the result of research? Research impact, and thus the benefits flowing from them is contextual and time dependent and involves a complex network of interrelated variables. Different stakeholders in research have different conceptions of the value and nature of research, its conduct, what it should achieve and the kinds of benefits expected. Therefore, there is no one-size fits all approach to measuring benefit; solutions to effectively combat benefit gaps cannot be homogenous across all levels of impact, but rather should be innovative and tailored to the situation. But this does not mean that the heterogeneity of context cannot align with standard areas of impact and measures. Australia currently uses a number of quality metrics to evaluate the value of research, but it is likely that benefit is going to assume an equal, if not greater importance in the future. We need to work collaboratively and listen more closely to the voice of Indigenous Australians to better understand, demonstrate and measure health research benefits. The authors conclude that as an imperative, a systematic benefit assessment strategy needs to be developed and implemented across research projects conducted with Indigenous partners. The authors of this article, supported and funded by collaboration with Lowitja Institute, are currently making progress in developing a benefit framework and step-by step guides to assist research stakeholders working in Indigenous Australian health to routinely plan, identify and monitor research benefit – a developmental paper of the process is forthcoming. This author team is also conducting two systematic searches of: 1) the total Indigenous health publication outputs to determine the extent to which researchers currently report the benefits of their research and how such benefits are framed; and 2) the evidence for approaches that can usefully guide and enhance thinking and practice to maximise Indigenous Australian health research impact and benefit. Concurrently, empirical research is being conducted to refine the preliminary benefit framework and articulate a clear definition of Indigenous Australian research benefit.

Academic and funding institutions will hopefully recognise that this kind of investment will bring wider benefits that can lead to additional funding opportunities and developing relationships that might enable subsequent research projects to achieve benefits aligned with Indigenous priorities and expectations. What is required is the development of new tools and thinking about what research benefit means - in Indigenous health research it will often mean adopting a benefit-led approach by changing the way research is done and preferencing of alternative research methodologies. The disconnecting agendas between researchers and Indigenous research partners have made for many missed opportunities; particularly that of research impact and benefit for all parties. While we do not yet have comprehensive answers to the questions proposed, we conclude that as a point of departure to improving impact and reaching mutually beneficial outcomes in Indigenous health research that we routinise the assessment of benefit from outset of research as one of the standards toward which we should work.
